# The evolution of left ventricular pseudoaneurysm from the rupture of left ventricular free wall following acute myocardial infarction: a case report

**DOI:** 10.1186/s12872-019-01321-2

**Published:** 2020-01-08

**Authors:** Xin Li, Yu Wang, Dong Wang, Chaohui Lai, Chenxin Wang

**Affiliations:** 1 Department of Cardiovascular, The Third Central Hospital of Tianjin, 83 Jintang Road , Hedong District, Tianjin, 300170 China; 2 Department of Ultrasonic, The Third Central Hospital of Tianjin, Tianjin, China; 3 Department of Respiratory medicine, The Third Central Hospital of Tianjin, Tianjin, China

**Keywords:** Pseudoaneurysm, Myocardial infarction, Percutaneous coronary intervention

## Abstract

**Background:**

Left ventricular pseudoaneurysm is a very rare complication following acute myocardial infarction, which results from a free wall rupture. Hemopericardium and cardiac tamponade caused by rupture of the free wall after acute myocardial infarction are often fatal. It is difficult to fully document the evolution of left ventricular pseudoaneurysm resulted from acute myocardial infarction with conservative treatment.

**Case presentation:**

Herein, we followed a 75-year-old female patient for 3 years. Recorded the evolution of the disease: acute lateral myocardial infarction - emergency reperfusion therapy - cardiac rupture - positive successful rescue - the pseudoaneurysm formation - maintaining conservative treatment - gradual enlargement of the pseudoaneurysm - thrombosis in pseudoaneurysm - thrombus filling with pseudoaneurysm - finally stabilized condition - the treatment of coronary revascularization.

**Conclusions:**

This case is reported here because of its scarcity, which provides provides us with a complete record of the entire evolution and an astonishing indication of the long-term prognosis of non-surgical treatment for pseudoventricular.

## Background

Left ventricular pseudoaneurysm is a very rare complication of acute myocardial infarction. Hemopericardium and cardiac tamponade caused by a free wall rupture following the acute myocardial infarction is often fatal. Only a few patients survive and develope left ventricular pseudoaneurysm. Owing to the possibility of death caused by subsequent complete rupture, surgical repair is usually recommended when a left ventricular pseudoaneurysm has been detected. However, we followed a 75-year-old female patient who had a left ventricular pseudoaneurysm for 3 years due to a ruptured left ventricular free wall after an acute myocardial infarction. We recorded the evolution of left ventricular pseudoaneurysm with conservative treatment.

## Case presentation

**Fig. 1:**
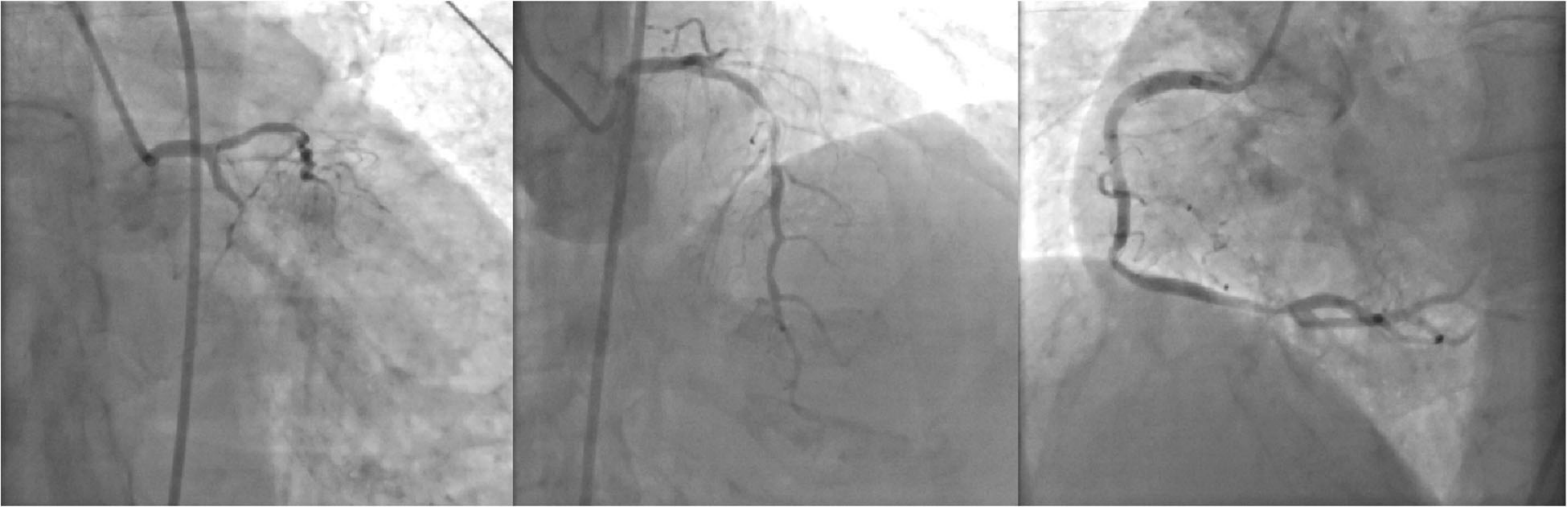
The CAG revealed that the patient present three-vessel coronary disease and the infarct-related artery is left circumflex artery (LCX)

A 75-year-old woman was admitted to our hospital for 1 h of persistent severe chest pain. Apart from smoking, she had no cardiac risk factors. At the time of admission, the electrocardiogram showed ST-segment elevation in leads I,avl. Laboratory data showed that the serum creatine kinase and troponin I had elevated. According to the results of electrocardiogram and laboratory data, STEMI was definitly diagnosed. Primary percutaneous coronary intervention (PCI) was indicated immediately. The coronary agiography (CAG) revealed triple vessel disease with left circumflex as the culprit which was occluded in the proximal segment (Fig. 1).

**Fig. 2:**
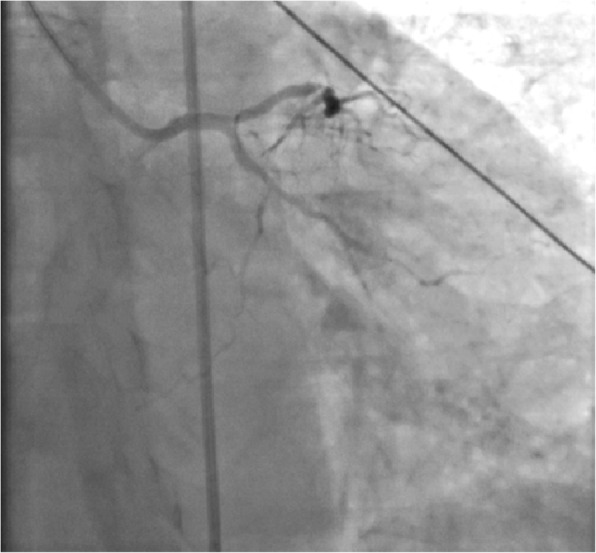
CAG after emergency PTCA showed revascularization of LCX

With the indication of aspiration thrombectomy and percutaneous coronary balloon dilatation, the coronary reperfusion therapy was achieved (Fig. 2). The symptom of chest pain was relieved. Meanwhile, the cardiac surgeon was invited for consultation, and his recommendation was to complete the CABG surgery after the acute phase of AMI. We ended the operation because the goal of primary percutaneous coronary intervention was achieved. The initial course went well, and the patient’s condition was stable gradually after PTCA. However, she suddenly exhibited severe chest pain, dyspnea, agitated, and profuse sweating 6 days after hospitalization. Physical examination indicated a blood pressure of 79/44 mmHg, a heart rate of 98 beats/minute. An electrocardiogram examination did not show ST-segment elevation again. She was given dopamine to raise the blood pressure and fluid to maintain the circulation capacity immediately. The patient presented cardiac syncope suddenly following the onset of atrial fibrillation with a heart rate of 140 beats per minute after half an hour. In consideration of further hemodynamic impairment caused by tachyarrhythmia, electrical defibrillation was urgently administered to restore sinus rhythm. Emergency echocardiography demonstrated pericardial tamponade. After pericardiocentesis with ultrasound guidance and continuous drainage of hemorrhagic pericardial effusion, the patient’s haemodynamic condition returned to stability. The catheter drainage of pericardium stopped when there was no fluid drainage in 2 days. Subsequent echocardiography revealed an abnormal cardiac chamber adjacent to the lateral wall of the left ventricle. Doppler echocardiography demonstrated to-and-fro signals in a myocardial defect (11 mm) in the lateral wall. And LVEF was 55%. These findings supported a diagnosis of congestive haemodynamic impairment caused by the rupture of the left ventricle after acute myocardial infarction followed by the presenting of acute pericardial tamponade. Timely pericardial drainage stabilized haemodynamics and pericardial adhesions sealed the rupture and contained the bleeding, and a pseudoaneurysm of the left ventricle formed eventually. Considering the tendency of rupture of pseudoaneurysm that may lead to death at any time, we recommended that the patient should receive surgical treatment as soon as possible. However, due to the resolute refusal and advanced age of patient, the conservative treatment was performed. Drug regimens included dual antiplatelet, statin, β-blocker and diuretics.

**Fig. 3:**
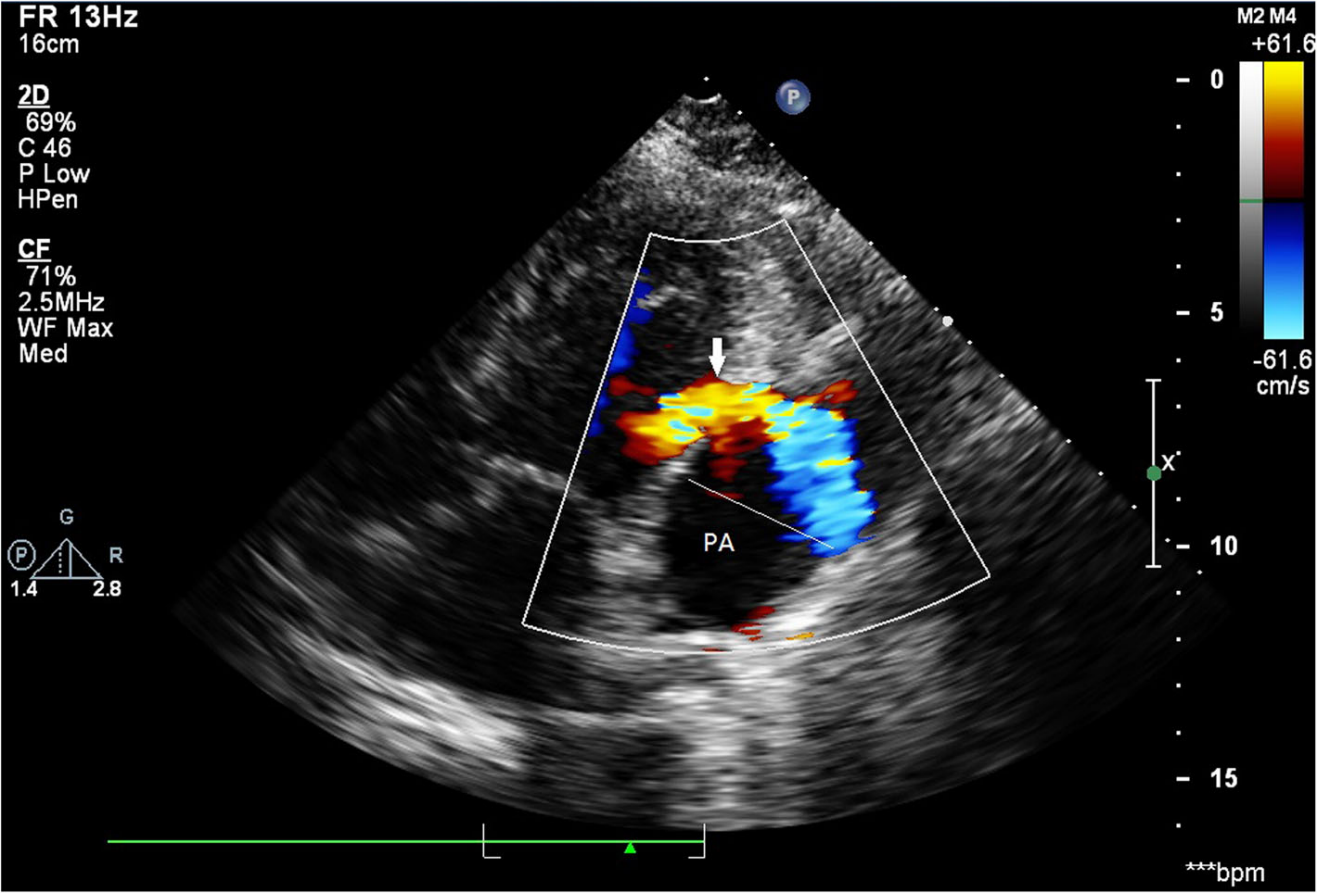
Doppler echocardiography demonstrated to-and-fro singals in a myocardial defect (11 mm) in the lateral wall (indicated by the arrow) and the formation of pseudoaneurysm (PA: pseudoaneurysm)

**Fig. 4:**
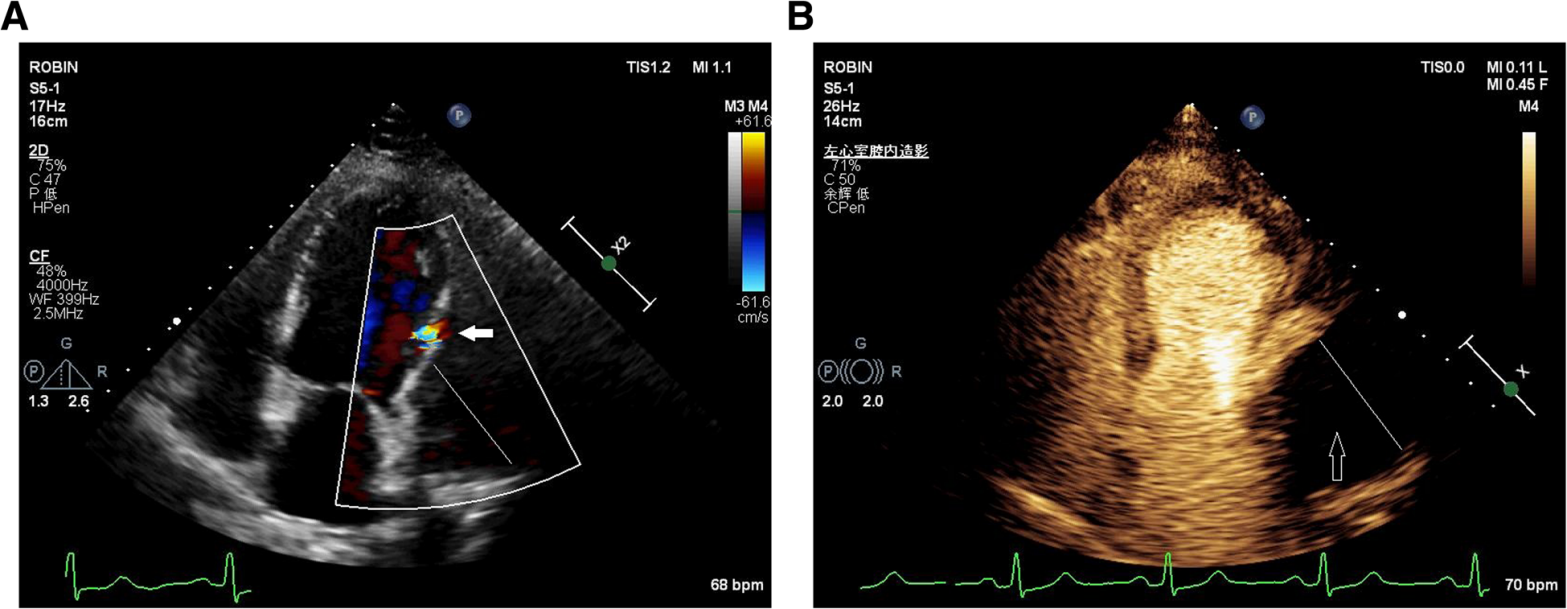
The picture **a** showed that the thrombus almost filled the aneurysm cavity. Partial blood flow imaging had been seen only nearby the defect of the left ventricular (indicated by the arrow). The picture **b** showed that myocardial contrast echocardiography clearly verified these findings. The black area (indicated by the arrow) without contrast agent filling suggested no blood flow into the pseudoaneurysm. It has been filled by thrombus completely

**Fig. 5:**
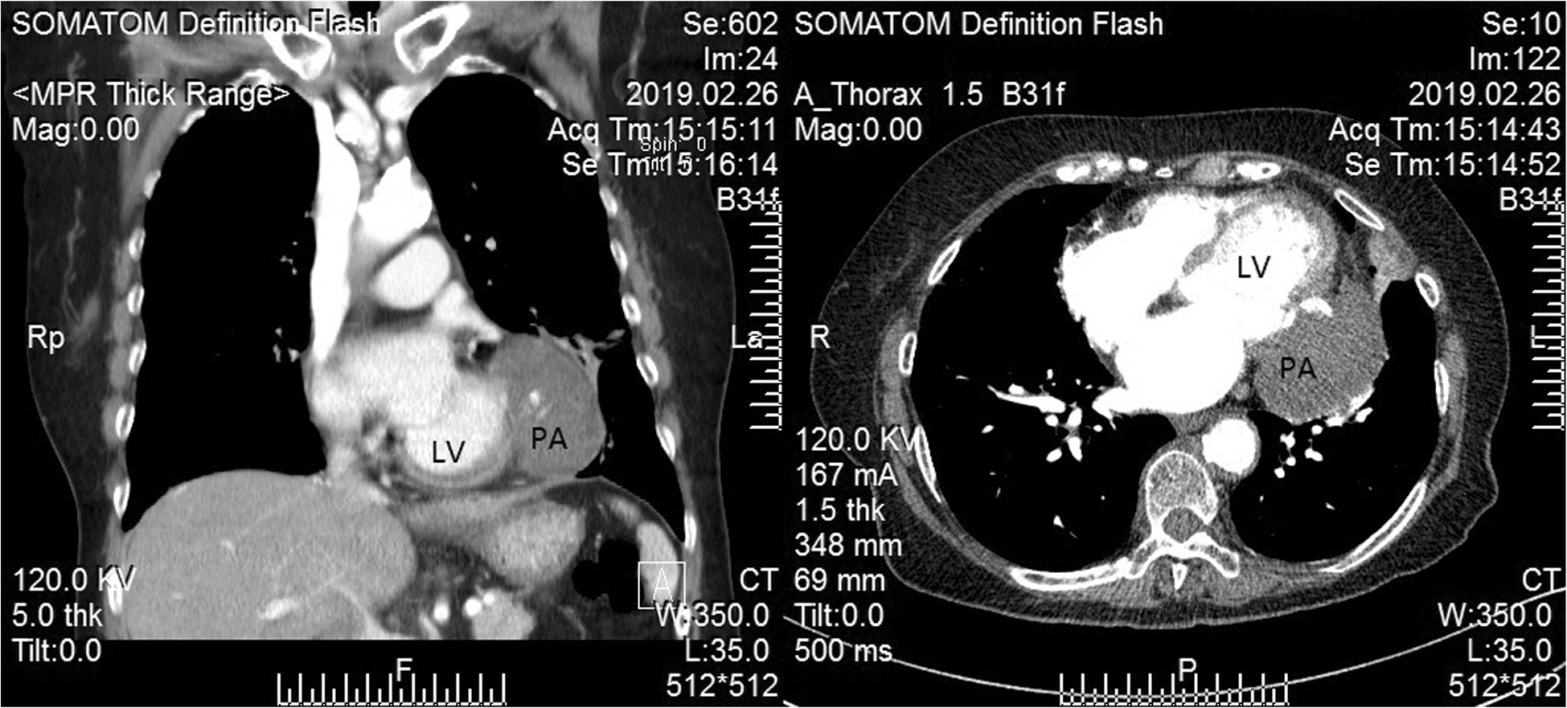
The result of Cardiac multislice computed tomography (MSCT) confiemed the formation of the pseudoaneurysm and thrombus filling with pseudoaneurysm. (PA: pseudoaneurysm)

**Fig. 6:**
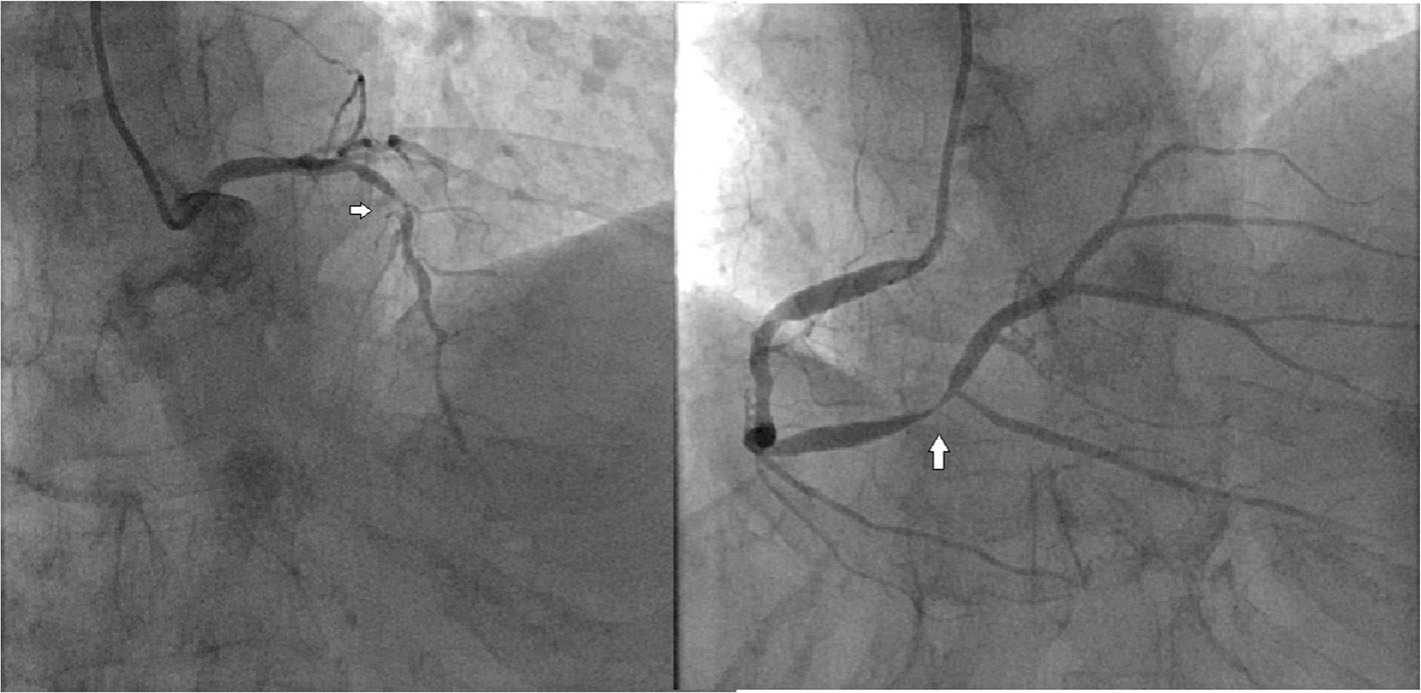
CAG revealed lesions in three branches of the coronary artery, with 90% stenosis in the middle segment of the left anterior descending (the arrow in the picture above) and 95% stenosis in the distal segment of the right coronary artery (the arrow in the picture below)

**Fig. 7:**
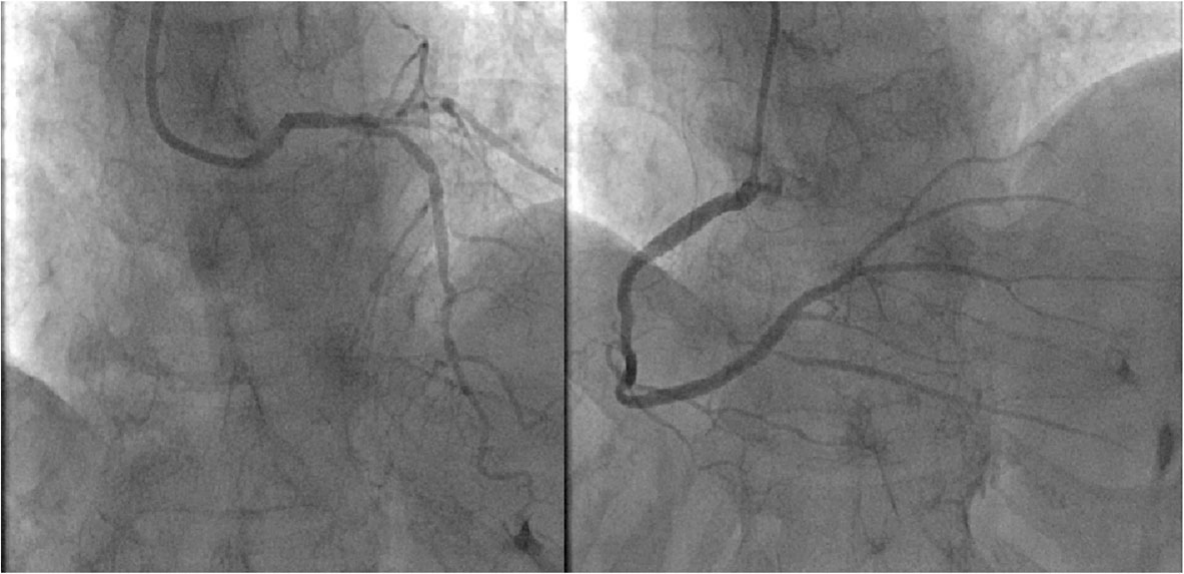
The CAG results after PCI showed improvement in the stenosis. Coronary revascularization has been completed

After discharge, the patient was followed up for a long time (36 months). During the first 2 months of follow-up, the volume of pesudoaneurysm increased to 65 × 55 mm(Fig. 3). Despite repeated warnings that pseudoaneurysm may further rupture causing sudden death, and recommendation for surgery as soon as possible, the patient persisted in conservative treatment. During this period, the patient’s condition was relatively stable, without obvious symptoms. However, an amazing change occurred in the following days of follow-up. One year after the onset of AMI, color doppler ultrasound revealed no progressive increase in the size of the pseudoaneurysm(53 × 44 mm), but the blood flow within the aneurysm cavity decreased(44 × 19 mm). Echoes of the thrombus had been seen in the other areas within the aneurysm. At the 1.5 years of follow-up, the thrombus almost filled the aneurysm cavity. Partial blood flow imaging had been seen only nearby the defect of the left ventricular. Further myocardial contrast echocardiography clearly verified these findings (Fig. 4). Because of the dynamic changes in the pseudoaneurysm showed that the risk of ventricular aneurysm rupture gradually reduced, we maintained the conservative treatment program. 2.5 years after the onset of AMI, she was admitted for angina once again. After admission, conventional color doppler ultrasound showed no significant change in pseudoaneurysm compared with 1 year ago. Cardiac multislice computed tomography (MSCT) clearly revealed a 64 × 48 mm pseudoaneurysm adjacent to the left ventricle. There was no contrast enhancement in it (Fig. 5). As angina pectoris manifested, coronary angiography was reviewed after admission. The result revealed lesions in three branches of the coronary artery, with 90% stenosis in the middle segment of the left anterior descending (LAD) and 95% stenosis in the distal segment of the right coronary artery (RCA) (Fig. 6). Therefore, LAD and RCA PCI were given, and one stent was implanted respectively (Fig. 7).

The symptoms of angina were significantly improved after operation. Our patient continued outpatient follow-up after discharge, and was symptom free. There was no significant change in medication during the 3 years of treatment. Dual antiplatelet therapy was continued for 3 years.

## Discussion and conclusion

Left ventricular free wall rupture is a very serious mechanical complication after AMI with high mortality rate. Myocardial rupture that directly causes death reportedly occurs in 4 to 10% of patients after acute myocardial infarction [1, 2]. Cardiac rupture after AMI generally occurs within the first week after its onset. And the rupture happens 90% possibility in the free wall. So the mortality rate after cardiac rupture is very high [3]. Acute pericardial tamponade following the ventricular wall rupture is also a common cause of death. Only a few of these patients passed the critical period and develpoed left ventricular pseudoaneurysm. In most reports, the pseudoaneurym body (epicardium) carries the risk of rupture at any time due to the continuous impacted of blood flow from the left ventricle. And ventricular pseudoaneurysm rupture, which may occur a long time after the myocardial infarction, is often induced by ischemia, fatigue, emotional agitation and with a high mortality rate [4, 5]. So most clinicians recommend surgical repair as soon as possible after diagnosis. Postoperative mortality after surgery of a left ventricular pseudoaneurysm ranges from 13 to 29% [6]. On the other hand, there are few long-term prognosis data for the conservative treatment of the left ventricular pseudoaneurysm due to the above reasons. However, in most of these reports, the outcome of patients with pseudoaneurysms treated conservatively have a poor prognosis. In one series, the mortality at 2 year is almost 50% [7].

In this case, the process of left ventricular pseudoaneurysm caused by rupture of left ventricular wall after myocardial infarction was clear. After a long time of follow-up, we were surprised to find that despite the high mortality, the disease could still be self-limiting for a period of time. Thrombus could form in the chamber of the aneurysm and increase gradually. However, it reduced the sustained impact of blood flow on the wall of the pseudoaneurysm. Eventually, the thrombus filled the aneurysm and prevented blood from flowing into the aneurysm from the left ventricular. The patient turned the corner. The dynamic evolution of this case demonstrated the feasibility and long-term benign prognosis of conservative treatment for left ventricular pseudoaneurysm.

In one study, 13% of the patients with a left ventricular pseudoaneurysm had systemic embolism as the clinical presentation [8]. In another study on the long term outcome of left ventricular aneurysm, the relatively high incidence of ischaemic stroke suggested that chronic anticoagulation should be considered in these patients [9]. Thrombus was also obviously observed in this case. However, the proliferation of thrombus results in a good prognosis for this patient. During the period of treatment, we did not use anticoagulant therapy in the expectation that the thrombus would fill up the pseudoaneurysm. We only used antiplatelet therapy (dual antiplatelet after stent implantation). Now the thrombus has almost filled the aneurysm completely, as we expected. We are now ready to initiate a combination of anticoagulant and monoclonal antiplatelet therapy to avoid the possible risk of thromboembolisim.

This is a rare case. First of all, timely and accurate treatment in the acute phase saved the life of this patient. Second, after a long period of follow-up, we have obtained the complete evolution of a left ventricular pseudoaneurysm: acute myocardial infarction - cardiac rupture - left ventricular pseudoaneurysm formation - gradual enlargement of the pseudoaneurysm - thrombosis in pseudoaneurysm - thrombus filling with pseudoaneurysm - condition recovery and stability. These data suggest that the evolution process and the long term outcome of the left ventricular pseudoaneurysm may be relatively benign after severe acute pericardial tamponade. Secondary thrombosis in the aneurysm, while increasing the risk of thromboembolism, might be a solution for the aneurysm to repair itself and reduce the risk of fatal rupture in the long term. Therefore, these suggest a possible benign endpoint for long-term prognosis after conservative treatment of left ventricular pseudoaneurysm.

## Data Availability

All available information is contained within the present manuscript.

## Abbreviations

AMI: Acute myocardial infarction
CABG: Coronary artery bypass graft
CAG: Coronary agiography
LAD: Left anterior descending
LCX: Left circumflex artery
MSCT: Cardiac multislice computed tomography
PCI: Percutaneous coronary intervention
PTCA: Percutaneous transluminal coronary angioplasty
RCA: Right coronary artery
STEMI: ST elevation myocardial infarction
